# Serum Urate Polygenic Risk Score Can Improve Gout Risk Prediction: A Large-Scale Cohort Study

**DOI:** 10.3389/fgene.2020.604219

**Published:** 2021-02-04

**Authors:** Yanfei Zhang, Ming Ta Michael Lee

**Affiliations:** Genomic Medicine Institute, Geisinger, Danville, PA, United States

**Keywords:** polygenic risk score, gout, serum urate, risk prediction, genomic medicine, precision health

## Abstract

Gout is a painful inflammatory arthritis affecting more than 8 million Americans. Identifying high-risk patients in early life could potentially encourage people to adopt lifestyle changes to prevent gout. Polygenic risk score (PRS) provides an overall estimate of an individual's genetic liability to develop a disease and can be used for early identification of high-risk individuals. In this study, we validated a previously reported PRS in an independent cohort. The urate-PRS was constructed from 110 significant urate-associated variants identified in Europeans. Phenome-wide and PRS-wide association study showed the urate-PRS is highly specifically associated with gout (phecode: 274.10; beta = 1.495 [1.372, 1.619], *p* = 4.37e-124). Urate-PRS alone did not performed in the gout prediction (area under the receiver operating characteristic curve, AUROC = 0.640); however, the addition of PRS upon demographics significantly improved the model performance, yielding an AUROC of 0.804 from 0.777 (DeLong test *p* = 3.66e−9). Trans-ethnic PRS and European-specific PRS showed similar prediction performance. We observed increasing gout prevalence and odds ratio (OR) across the PRS quintiles. Our study showed 8.2% of the cohort had more than 2.5 odds for gout than remainders, indicating that urate-PRS may be a better marker than age and sex to stratify patient risk. With the rapid growth of large biorepositories, such as All of Us, urate-PRS can be applied quickly and widely in population to estimate individual's risk, providing a powerful tool for gout preventive purpose in population health.

## Introduction

Gout is the most common inflammatory arthritis affecting more than 8 million Americans, representing a significant cause of morbidity and healthcare costs (Dalbeth et al., 2016). It is an inflammatory response to the deposition of monosodium urate crystals that usually associated with elevated serum urate concentrations. Risk factors for gout include age, sex, obesity, diet, and genetics (Dalbeth et al., 2016). A recent large-scale trans-ethnic genome-wide association study (GWAS) meta-analysis by CKDGen consortium identified 183 urate-associated loci (Tin et al., 2019). Such large-scale GWAS meta-analysis provided the foundation for translational research of polygenic risk score (PRS).

PRS provides an overall estimate of an individual's genetic liability to develop a disease. As it is based on germline DNA, PRS holds the advantage for early risk screening and primary prevention over some conventional risk factors such as age and sex (Torkamani et al., 2018). Given that gout attacks are painful and the current challenges in gout management, such as a variety of different urate-lowering medications and the absence of clinical trial of long-term benefit of treat-to-target strategy (Perez-Ruiz and Dalbeth, 2019), prevention is an important strategy for gout management. Identifying patients at high risk for gout using urate-PRS or gout prediction models could potentially encourage people to adopt lifestyle changes that reduce the risk factors, such as obese and diet, to prevent gout. A PRS constructed from 114 significant urate-associated variants identified in Europeans showed promising result to improve gout risk prediction in the UK Biobank dataset (Tin et al., 2019). However, validation in an independent cohort is warranted before clinical implementation.

Geisinger is a learning healthcare system in central and northeast Pennsylvania. The MyCode Community Health Initiative (MyCode) is an ongoing research project that holds de-identified electronic health record (EHR) linked with genetic data (Carey et al., 2016; Dewey et al., 2016). Currently, over 266,000 participants have been consented to participant in MyCode. Such project provides amber resources for genetic studies aiming at discovery, translational, and precision health research. The aim of our study is to validate the utility of urate-PRS in an independent cohort by leveraging the EHR and linked genetic data of MyCode.

## Method

### MyCode Cohort and Genetic Data

The study cohort included participants from MyCode phase I (*N* = ~60,000) and phase II (*N* = ~32,000). All the MyCode participants provided consent to allow their EHR and genetic data to be used for research (Carey et al., 2016). Samples were genotyped using Illumina Infinium OmniExpress Exome array and GSA-24v1-0 array for phase I and II, respectively. Genotypes for both cohorts were imputed to HRC.r1-1 EUR reference genome (GRCh37 build) separately using the Michigan Imputation Server. Filters applied on the central dataset include info score >0.3, sample missingness <5%, marker missingness <5%, and Hardy–Weinberg equilibrium (*p* > 1e-7). In this study, we only included participants of European ancestry. We also removed one of the first- or second-degree related pair of participants with PI_HAT ≥ 0.125. Supplementary Figure 1 illustrates the principal component analysis plot of the cohort. This study received an exemption from the Geisinger Institutional Review Board for using de-identified data. We obtained approval from the MyCode Governing Board to do the genetic study.

### Urate Polygenic Risk Score Calculation

We obtained 110 of the 114 urate-associated single-nucleotide variants (SNVs) with genome-wide significance in Europeans from the serum urate GWAS meta-analyses (Supplementary Table 1) (Tin et al., 2019). Four variants were not included because of poor imputation quality. We also constructed a PRS using 172 out of 183 genome-wide significant index SNVs identified from trans-ethnic meta-analysis (Supplementary Table 2) (Tin et al., 2019). Eleven SNVs were not included due to poor imputation quality. The PRS was calculated as a weighted sum of the effect allele using PLINK1.9 (Chang et al., 2015).

### Phenome-Wide Association Study

The demographic data and diagnosis history for each participant were retrieved from the de-identified EHR database at Geisinger. Diagnosis was structured using International Classification of Diseases, Clinical Modification codes 9th edition (ICD-9). ICD-9 codes were mapped to a standardized phecode using the map version v1.2 (Denny et al., 2013). A phecode matrix was built first, where we defined a case should have a phecode at ≥2 different encounters and a control should not have the same or related phecodes. Individual with phecode at only one encounter was neither a case nor a control. Phecodes with case number ≥40 were included in the phenome-wide association study (pheWAS). Finally, 1,437 phecodes and 45,351 participants were included in the final phecode matrix for subsequent PheWAS. We employed a logistic regression model adjusted for age, sex, BMI, and the first 10 genetic principal components (PCs) to control the potential population structure. Bonferroni significance is defined *p* <3.48e−5 (0.05/1,437).

### PRS-Wide Association With Gout

To validate the specificity of urate-PRS with gout, we constructed a list of established PRS for other traits and diseases and tested their association with gout. We extracted the weight score file from The Polygenic Score (PGS) Catalog database (https://www.pgscatalog.org). Supplementary Table 3 lists the details of PRS for BMI (Khera et al., 2019), low-density lipoprotein cholesterol (LDL) (Trinder et al., 2020), type 2 diabetes (T2D), atrial fibrillation, coronary heart disease (CHD), breast cancer, prostate cancer (Mars et al., 2020), and inflammatory arthritis (Knevel et al., 2020) including rheumatoid arthritis, psoriatic arthritis, and spondyloarthropathy. Logistic regression adjusted for age, sex, BMI, and the first 10 PCs to test the association of the PRS with gout. Bonferroni significance is defined at *p* < 0.005.

### Odds Ratio of PRS for Gout

We adopted a “vs. remainders of the population” method to evaluate the odds ratio (OR) of PRS (Khera et al., 2018). Specifically, individuals with the PRS that is greater than a threshold are considered as “carriers” and the remainder population are considered as “non-carriers”. Gout cases (*N* = 2,929) and controls (*N* = 41,133) were extracted from the phenome matrix using a parent-level phecode “274” to maximize the case sample size. A logistic regression model adjusted for age, sex, and BMI was used to evaluate the OR for PRS > threshold. We scanned a range of PRS thresholds to identify the percentage of population that have an OR ≥ 3 and 2.5. We also divided PRS into quintiles. The number of gout and total individuals were counted. Using the most common 3rd quintile as reference, we calculated the age, sex, and BMI-adjusted ORs across PRS quintiles using logistic regression.

### Risk Prediction Models of Gout

As the MyCode phase I samples were collected first, we used the phase I data as the training dataset (2,368 case and 29,530 controls) and the phase II data as the testing dataset (561 case and 11,603 controls). It should be noted that a subset of samples from phase I who had serum urate values was part of the consortium GWAS meta-analyses (Tin et al., 2019). However, this won't affect building the models in training dataset, as identification of the urate-associated variant can be considered as feature selection, an important step in model construction (Dankers et al., 2019). Logistic regression models were used to regress gout on the (1) urate-PRS alone; (2) age, sex, and BMI; and (3) combined PRS with age, sex, and BMI in the training dataset. Each of the three models was then used to predict gout status in the testing samples. Model performance was evaluated using the area under the receiver operating characteristic curve (AUROC). Ten-fold cross-validation was used to estimate the mean and standard deviation of the AUROC of the three models in the training dataset. As a logistic regression calculate the log(odd) or the probability of each sample belonging to the case category, a cutoff threshold is needed to predict the gout status. We identified the best threshold by maximizing the Youden's index (sensitivity + specificity – 1) in the training dataset. We then used this threshold to predict the gout status in the testing dataset and report the sensitivity and the specificity. We also examined a urate-PRS constructed using significant SNVs identified from trans-ethnic meta-analysis as described above.

## Results

### High Specificity of Urate-PRS for Gout Indicated by PheWAS

**Figure 1:**
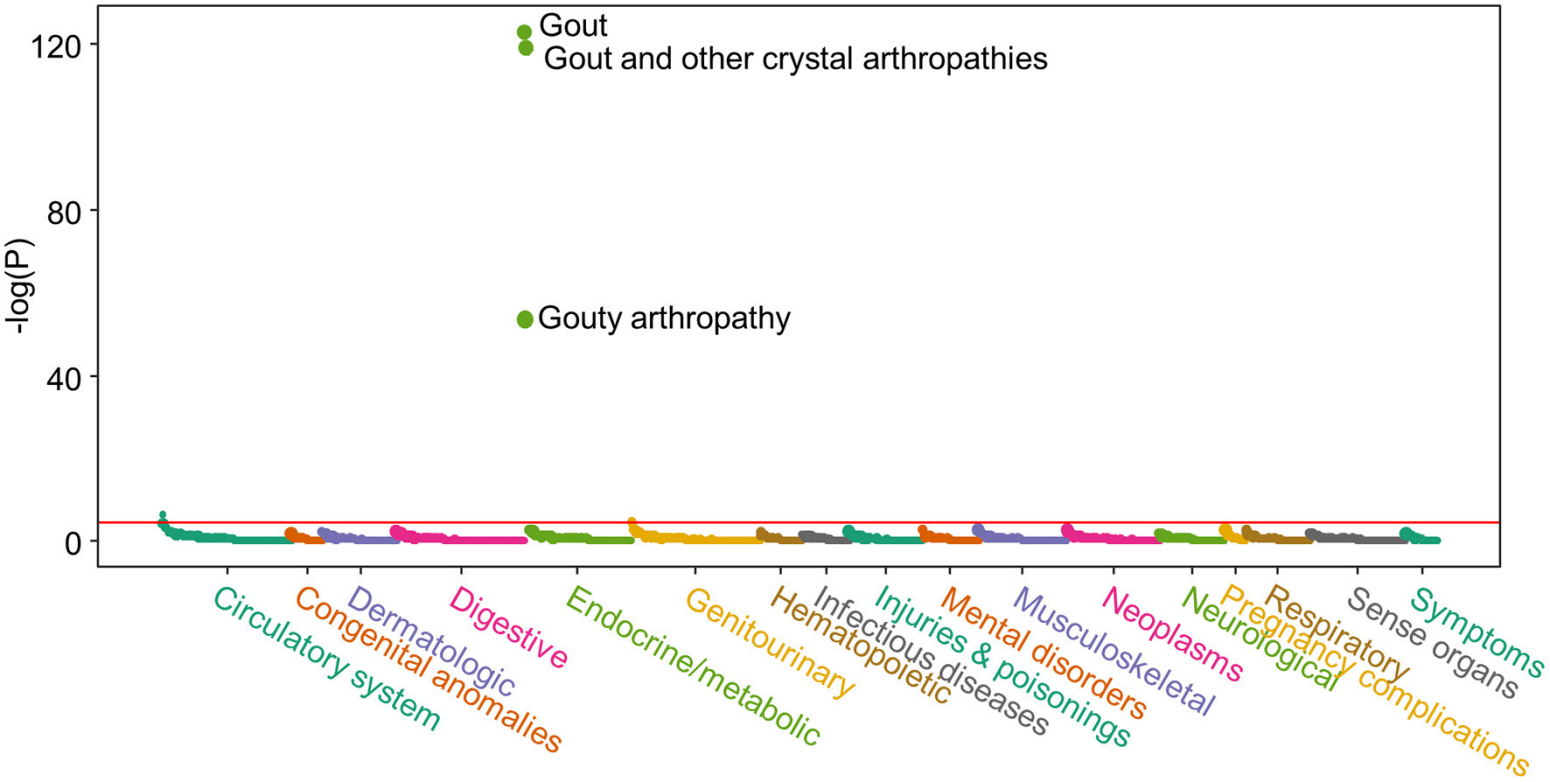
Manhattan plot of the polygenic risk score (PRS)-phenome-wide association study (PheWAS) result. X-axis represents each phenotype; Y-axis represents the negative logarithm of the raw *p*-value of PRS term in the logistic regression model. Horizontal line indicates Bonferroni significant level (*p* = 3.48e-5). Logistic regression model was adjusted for age, sex, BMI, and the first 10 genetic principal components to control the potential population structure. Supplementary Table 4 lists the results for phenotypes with *p* < 0.05.

We first performed a PRS-PheWAS to show the validity of the urate-PRS. Figure 1 shows the Manhattan plot for the PRS-PheWAS results. The urate-PRS showed specific and significant association with gout [phecode: 274.10; beta (95% confidence interval, CI) = 1.495 (1.372, 1.619), *p* = 4.37e−124], gout arthropathy [phecode: 274.11; beta (95% CI) = 1.664 (1.454, 1.875), *p* = 3.24e−54], gout and other crystal arthropathies [phecode: 274; beta (95% CI) = 1.453 (1.331, 1.575), *p* = 3.96e−120]. Other phecodes that approached the Bonferroni significance level include essential hypertension (phecode: 401 and 401.1) and chronic kidney disease (phecode: 585.33 and 585.34), all of which increase gout risk but showed much less significant than the association with gout (Figure 1 and Supplementary Table 4 for phenotypes with *p* < 0.05), indicating the specificity of the urate-PRS for gout. To further validate the specificity, we examined the associations of PRS of other traits and diseases with gout (Supplementary Table 5). We observed significant associations of gout with PRS of atrial fibrillation [beta (95% CI) = 1.040 (0.570, 1.509), *p* = 1.42e−5], type 2 diabetes [beta (95% CI) = 0.733 (0.368, 1.097), *p* = 8.17e-5], and coronary heart disease [beta (95% CI) = 0.928 (0.446, 1.410), *p* = 1.63e−4]. However, urate-PRS has much larger magnitude of the significance and effect for gout association (1e-120 vs. 1e-5).

### Odds Ratio of the Urate-PRS for Gout

**Figure 2:**
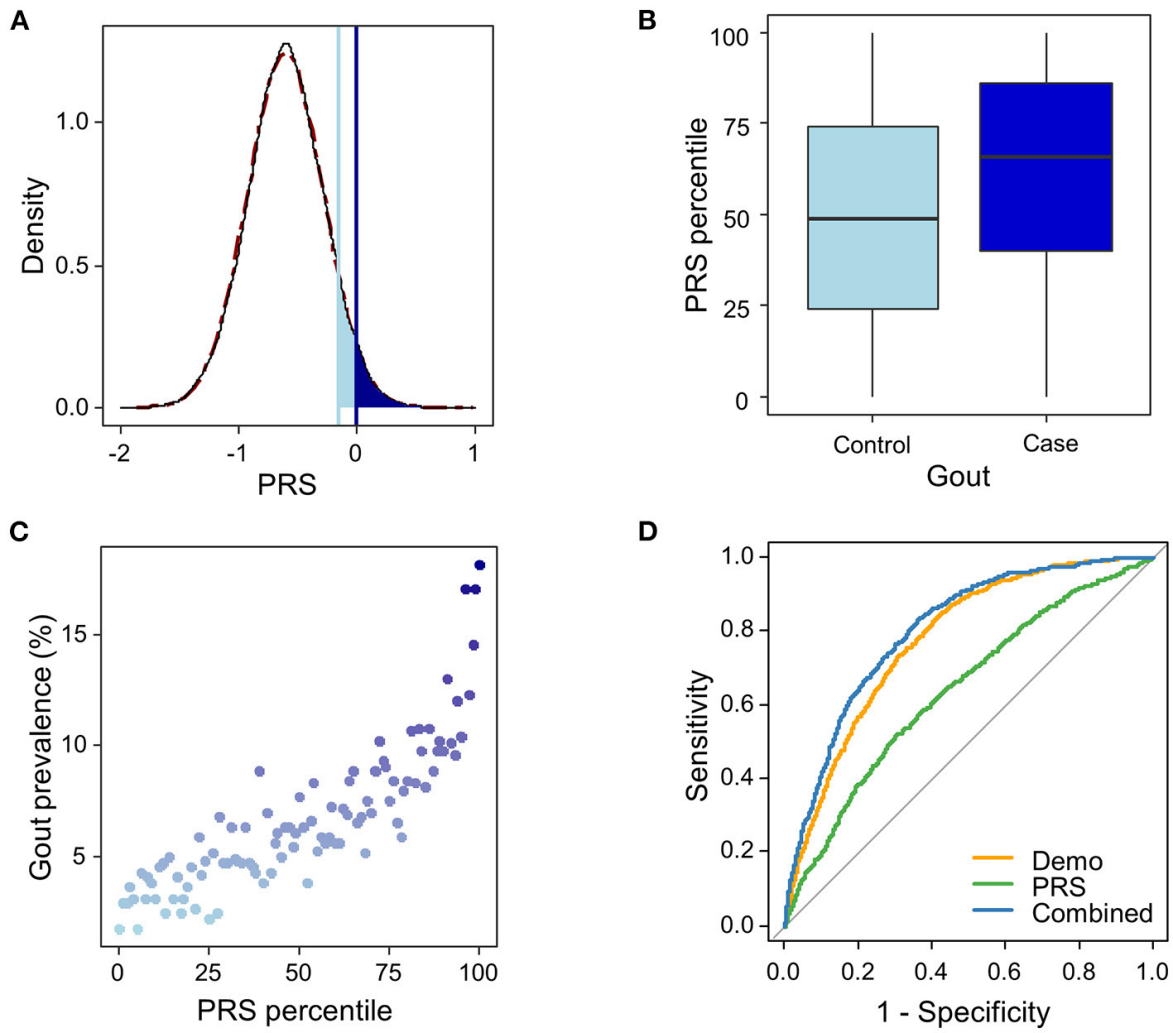
Risk and prediction for gout by urate-polygenic risk score (PRS) derived in European ancestry. **(A)** Distribution of PRS (*n* = 44,062). A theoretical normal distribution (dashed line in dark red) was overlaid on the PRS density plot. Shading reflects the proportion of the population with 2.5 (light blue) and 3-fold (dark blue) increased risk vs. the remainder of the population. The odds ratio was assessed in a logistic regression model adjusted for age, sex, BMI, and genotyping array. **(B)** PRS percentile among gout cases vs. controls. Within each boxplot, the horizontal lines reflect the median, the top and bottom of each box reflect the interquartile range, and the whiskers reflect the maximum and minimum values within each grouping. **(C)** Prevalence of gout in 100 groups binned according to the percentile of the PRS. **(D)** The ROC curves in the testing dataset from the three logistic regression models trained in the training dataset using PRS only (green line), demographic features (age, sex, and BMI; orange line), and the combined features (blue line).

The urate-PRS is normally distributed across the population (Figure 2A). Gout cases showed higher PRS than the controls, with a median PRS percentile of 66 for gout cases vs. 49 for the controls (*t*-test *p* <2.2e-16, Figure 2B). The prevalence of gout rises sharply in the right tail of the distribution, from 1.8% in the lowest percentile to 18.1% in the highest percentile (Figure 2C). To assess the effect of PRS on gout risk, we adopted a “vs. remainders” way to test the odds ratio of a PRS that is greater than a threshold (Khera et al., 2018). We observed 3.0% (PRS > 0.001) of the population had OR ≥ 3, an effect comparable to variants with a modest Mendelian effect size (Richards et al., 2015), compared to the remainders of the population (Figure 2A). We also observed that 8.2% (PRS > −0.16) of the population had OR ≥ 2.5 compared with the remainders (Figure 2A).

**Figure 3:**
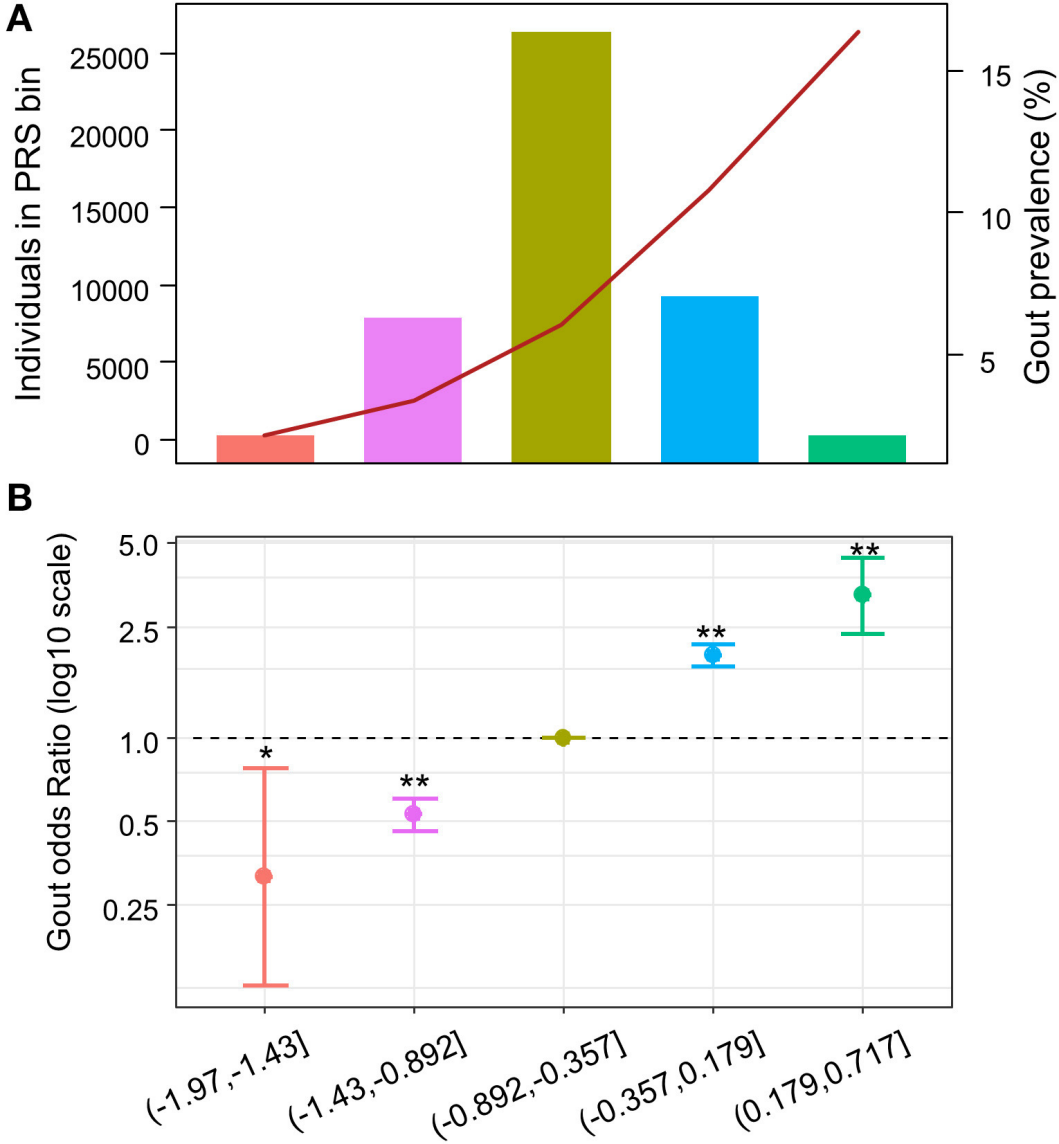
Odds ratio across polygenic risk score (PRS) quintiles. X-axis indicates the PRS quintile. **(A)** Number and gout prevalence across urate-PRS quintiles in 44,062 unrelated participants of European ancestry. Bar plot and left y-axis indicate the number of individuals in each bin; line plot and right y-axis indicate the gout prevalence. **(B)** Age, sex, and BMI-adjusted OR of gout (y-axis) across PRS quintiles. The third quintile was used as reference. The dots indicate mean OR; the bars indicate the 95% CI. *p < 0.05, **p < 5e−10.

To evaluate whether patients with higher PRS have higher odds for gout, we divided the PRS into quintiles. Similarly, we observed increasing gout prevalence across the PRS quintile bins, from 2.2 to 16.4% (Figure 3A and Supplementary Table 6). Using individuals in the 3rd quintile PRS bin, which contains most individuals among all PRS bins, as reference population, we calculated ORs for patients in other PRS bins to develop gout adjusting for age, sex, and BMI. We observed increasing ORs across PRS quintile bins (Figure 3B and Supplementary Table 6). The patients in the highest PRS bin had an OR of 3.23 (95% CI = 2.35–4.44, *p* = 4.52e-13).

### Addition of Urate-PRS in Gout Prediction Model Improves Performance

To evaluate the potential utility of the PRS in gout risk prediction, we constructed three logistic regression models using (1) urate-PRS; (2) age, sex, and BMI; and (3) all variables in the training dataset. Ten-fold cross-validation of the logistic models in the training dataset provided mean AUROCs of 0.62 (SD = 0.020), 0.75 (SD = 0.016), and 0.77 (SD = 0.018) for the three models, respectively. The models trained on the whole training dataset were then used to predict the gout status in the testing dataset. The model using only PRS performed weaker prediction (AUROC = 0.640) than the model using demographic features (AUROC = 0.777) or the model using combined features (AUROC = 0.804; Figure 2D, Table 1). Addition of urate-PRS in the model significantly increased prediction performance (DeLong test *p* = 3.66e−9). We identified the cutoff threshold for the logistic regression classifier by maximizing the Youden's index (sensitivity + specific – 1) in the training dataset. The best log odd threshold is −2.58 achieving a sensitivity of 75.5% and a specificity of 65.9% in the training dataset. Using this threshold, we obtained a sensitivity of 69.3% and a specificity of 75.5% in the test dataset (Supplementary Table 7).

**Table 1:** Prediction performance of three models and two urate-PRS.

**Model**	**European urate-PRS**		**Trans-ethnic urate-PRS**	
	**AUROC**	**DeLong test**	**AUROC**	**DeLong test**
PRS	0.640		0.630	
Demographics	0.777		0.777	
Combined	0.804	3.66e−9	0.801	8.08e−8

*European urate-PRS is calculated from 110 of the 114 independent variants from meta-analysis of European populations. Trans-ethnic urate-PRS is calculated from 172 of the 183 variants from the trans-ethnic meta-GWAS for urate acid. Demographics model includes age, sex, and BMI. AUROC, area under the receiver operating characteristic curve; PRS, polygenic risk score*.

We wonder whether PRS calculated using 183 variants identified from trans-ethnic meta-GWAS can perform better than PRS calculated using variants identified from meta-analysis of European analysis. We performed the same analysis for trans-ethnic urate-PRS. We observed an AUROC of 0.630 for the model using only PRS and an AUROC of 0.801 for the combined model (Table 1). Addition of the trans-ethnic PRS also significantly improved the prediction (DeLong test *p* = 8.08e-8). However, there is no significant difference using either European urate-PRS or trans-ethnic-PRS (DeLong test *p* = 0.358), although AUROC for models using European PRS are slightly better (Table 1).

## Discussion

Large-scale GWAS have discovered the polygenic genetic architecture for most of the traits, providing the foundation for constructing polygenic risk scores. In this study, we constructed a PRS from 110 variants that are significantly associated with serum uric acid in European ancestry. PheWAS of the urate-PRS and PRS-wide association with gout showed that urate-PRS is highly specific and significantly associated with gout or gout-related phecode. Individuals with gout had higher PRS than controls. We observed increasing gout prevalence and ORs across PRS quintiles. According to our analysis, 3% of the study cohort had OR > 3 than the remainders to develop gout and 8.2% had OR > 2.5. Urate-PRS alone performed poorly in the gout prediction. However, the addition of PRS can significantly improve the performance, yielding an AUROC of 0.804.

PheWAS showed that the urate-PRS is highly specific. It is highly significantly associated with gout-associated phecodes but no other phecodes, indicating it may be a potential good marker for gout prediction. In the subsequent evaluation of gout prediction models using PRS only, demographic features only, and the PRS with demographic features, we found that PRS itself is a weaker predictor. However, addition of the PRS in the model can significantly improve the model performance. Our results are comparable to the previously reported results using the UK Biobank data (Tin et al., 2019). Using the best estimate of the log odd cut-off value, we tested the gout prediction in the testing dataset. The model using PRS combined with demographic features can correctly identify 69% of all cases; however, it should be noted that the positive prediction value of the model is low (12.0%), indicating a low discriminative ability and thus could not be used for diagnostic purpose. On the other hand, as the PRS represents a lifelong genetic predisposition to higher urate levels and is possible to be calculated at birth, it may be valuable to stratify a group of patients with high risk for gout. We observed increasing prevalence and ORs across PRS quintiles. Our study showed 8.2% of the cohort had more than 2.5 odds for gout risk than the remainders, indicating that urate-PRS may be a better marker than age and sex to identify patients with high risk. With the growing of population- and healthcare system-based biorepositories, such as the ongoing project All of US, urate-PRS can be applied quickly and widely in population to estimate individual's risk. Urate-PRS may be a powerful tool for gout preventive purpose in healthy population, and it allows people to know their risk in early life and to choose a compensatory lifestyle to reduce the risk of gout in the future.

One limitation of our study is the lack of ancestry diversity. Majority of our cohort are of European ancestry; thus, we do not know how the PRS performs in population with other ancestries. PRS constructed using variants identified from populations with European ancestry may perform poorer in populations with a different ancestry (Duncan et al., 2019). Thus, large-scale GWAS in individuals with different ancestry other than European are required to build a powerful PRS before its implementation in populations with other ancestries. We also examined the performance of PRS derived from the trans-ethnic meta-analysis. The AUROC of the combined model using trans-ethnic PRS was very similar to the one using European PRS, suggesting that ancestry-specific PRS may perform equally well as the trans-ethnic PRS when the sample size of GWAS meta-analysis is large enough. However, trans-ethnic PRS was reported to improve risk prediction in diverse populations (Marquez-Luna et al., 2017).

In conclusion, we validated that the urate-PRS is highly specific and can significantly increase the performance of gout prediction in an independent cohort. The urate-PRS and gout prediction model can be valuable for preventive but not diagnostic purpose in healthy population. Future prospective studies are needed to validate the potential benefit of early identification of high-risk individuals using PRS and early intervention in lifestyle and diet for gout prevention.

## Data Availability Statement

The data analyzed in this study is subject to the following licenses/restrictions: Access to the genetic and electronic health record data used in this study requires collaboration and data use agreement with author's institute. Please contact the corresponding author. Requests to access these datasets should be directed to Yanfei Zhang, yzhang1@geisinger.edu.

## Author Contributions

All authors listed have made a substantial, direct and intellectual contribution to the work, and approved it for publication.

## Conflict of Interest

The authors declare that the research was conducted in the absence of any commercial or financial relationships that could be construed as a potential conflict of interest.
