# Supplementary material for: Serum Urate Polygenic Risk Score Can Improve Gout Risk Prediction: A Large-Scale Cohort Study
Source: Front Genet. 2021 Feb 4;11:604219. doi: 10.3389/fgene.2020.604219 (PMC7889590; doi:10.3389/fgene.2020.604219)
Supplement: Supplementary file 1 [file Table_1.DOCX]

## Supplementary figure 1.


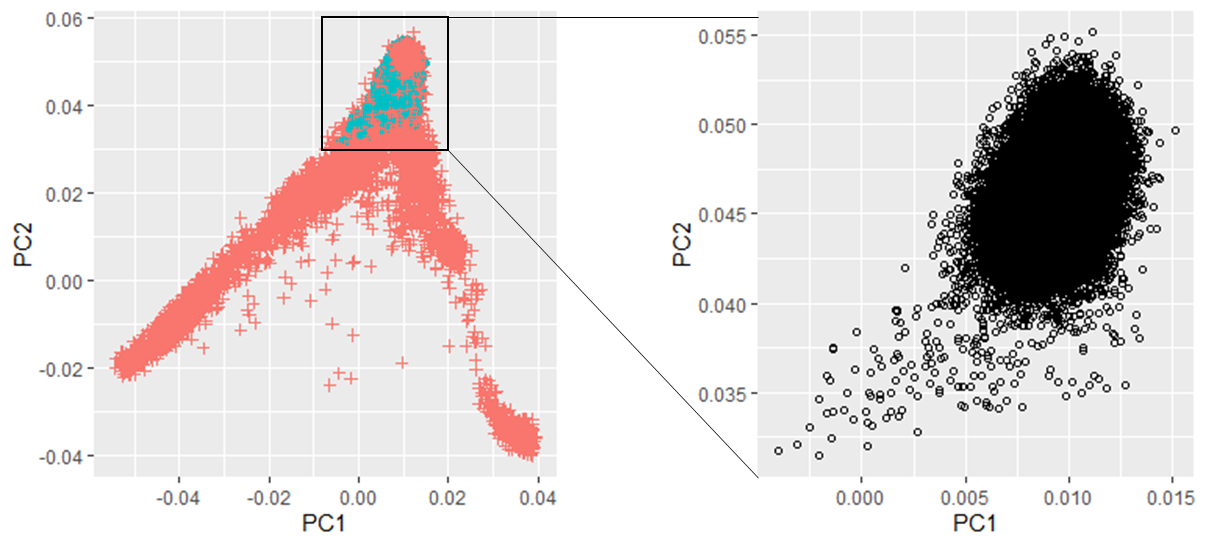


Supplementary figure 1. PCA plot for the MyCode cohort (left panel). Only unrelated participants of European ancestry were included (cyan dots in left panel, right panel) in the analysis. A very small portion of participants are outside of the main cluster (right panel), but there are no particular indications to remove them. Adjustment of the first 10 PCs will account for the potential population structure.

## Supplementary table 1. SNPs derived from GWAS of European ancestry used to calculate the urate-PRS

| SNV | Chr | Position(b37) | Nearest gene | EA | OA | EAF | Weight | p-value |
| --- | --- | --- | --- | --- | --- | --- | --- | --- |
| rs6825187 | 4 | 9,915,325 | *SLC2A9* | T | C | 0.357 | 0.187 | 0 |
| rs10017305 | 4 | 10,401,223 | *ZNF518B* | T | C | 0.709 | 0.214 | 0 |
| rs1481012 | 4 | 89,039,082 | *ABCG2* | A | G | 0.889 | -0.249 | 0 |
| rs1359232 | 6 | 25,809,716 | *SLC17A1* | A | C | 0.466 | -0.091 | 4.12E-126 |
| rs71456318 | 11 | 64,332,862 | *SLC22A11* | A | C | 0.484 | 0.079 | 4.41E-92 |
| rs1260326 | 2 | 27,730,940 | *GCKR* | T | C | 0.398 | 0.070 | 4.61E-69 |
| rs1171617 | 10 | 61,467,182 | *SLC16A9* | T | G | 0.768 | 0.079 | 1.81E-66 |
| rs12313306 | 12 | 57,751,854 | *R3HDM2* | T | C | 0.246 | -0.076 | 6.74E-65 |
| rs73224492 | 4 | 10,440,925 | *ZNF518B* | A | G | 0.875 | -0.094 | 9.00E-59 |
| rs10910845 | 1 | 145,723,120 | *NBPF20* | A | C | 0.469 | 0.058 | 1.50E-51 |
| rs2022051 | 11 | 64,367,589 | *SLC22A12* | A | G | 0.793 | -0.070 | 5.13E-48 |
| rs12530084 | 6 | 7,214,676 | *RREB1* | T | C | 0.220 | 0.066 | 9.55E-48 |
| rs2622629 | 4 | 89,094,064 | *ABCG2* | T | C | 0.636 | -0.057 | 8.42E-44 |
| rs2070803 | 1 | 155,157,715 | *TRIM46* | A | G | 0.578 | 0.053 | 4.09E-41 |
| rs10994860 | 10 | 52,645,424 | *A1CF* | T | C | 0.180 | 0.064 | 1.31E-36 |
| rs2581817 | 3 | 53,071,797 | *SFMBT1* | C | G | 0.420 | 0.048 | 4.87E-35 |
| rs10896028 | 11 | 65,432,187 | *RELA* | A | T | 0.645 | -0.048 | 4.10E-33 |
| rs10851885 | 15 | 76,304,503 | *NRG4* | A | G | 0.756 | -0.054 | 4.16E-32 |
| rs12908437 | 15 | 99,287,375 | *IGF1R* | T | C | 0.376 | 0.046 | 1.56E-30 |
| rs2943539 | 8 | 76,479,839 | *HNF4G* | T | C | 0.475 | 0.041 | 6.42E-28 |
| rs10223666 | 6 | 43,805,502 | *VEGFA* | C | G | 0.704 | 0.046 | 6.62E-28 |
| rs13226650 | 7 | 73,017,005 | *MLXIPL* | A | G | 0.809 | 0.049 | 1.35E-23 |
| rs9895661 | 17 | 59,456,589 | *BCAS3* | T | C | 0.817 | 0.050 | 7.23E-23 |
| rs3794748 | 17 | 53,365,172 | *HLF* | A | G | 0.409 | 0.038 | 1.38E-21 |
| rs34861762 | 8 | 23,748,420 | *STC1* | T | C | 0.419 | 0.034 | 3.50E-19 |
| rs1649078 | 10 | 60,293,320 | *BICC1* | A | C | 0.480 | -0.039 | 4.09E-19 |
| rs62052820 | 16 | 69,575,238 | *MIR1538* | A | G | 0.212 | 0.041 | 2.81E-18 |
| rs57652769 | 16 | 79,753,976 | *MAFTRR* | T | C | 0.309 | -0.036 | 8.56E-18 |
| rs9287911 | 2 | 170,037,294 | *LRP2* | A | T | 0.250 | 0.038 | 1.13E-17 |
| rs10774625 | 12 | 111,910,219 | *ATXN2* | A | G | 0.483 | 0.032 | 5.54E-17 |
| rs28530689 | 12 | 122,500,748 | *LOC100506691* | A | C | 0.512 | 0.032 | 1.27E-16 |
| rs62435145 | 7 | 1,286,567 | *UNCX* | T | G | 0.689 | 0.042 | 2.36E-16 |
| rs62128132 | 19 | 50,217,955 | *CPT1C* | T | C | 0.966 | -0.118 | 1.32E-15 |
| rs17050272 | 2 | 121,306,440 | *LINC01101* | A | G | 0.421 | 0.032 | 1.57E-15 |
| rs3925584 | 11 | 30,760,335 | *DCDC1* | T | C | 0.552 | 0.030 | 1.66E-15 |
| rs9925837 | 16 | 79,927,303 | *LINC01229* | A | G | 0.845 | -0.042 | 5.85E-15 |
| rs79598313 | 1 | 27,284,913 | *KDF1* | T | C | 0.026 | 0.100 | 9.22E-15 |
| rs11204701 | 1 | 150,662,179 | *GOLPH3L* | A | T | 0.779 | -0.036 | 1.05E-14 |
| rs4897160 | 6 | 126,223,944 | *NCOA7* | A | G | 0.483 | 0.030 | 1.96E-14 |
| rs1574430 | 6 | 43,269,029 | *SLC22A7* | A | C | 0.405 | 0.029 | 2.77E-14 |
| rs10971420 | 9 | 33,125,000 | *B4GALT1* | T | C | 0.688 | 0.031 | 4.14E-14 |
| rs57737646 | 15 | 76,299,828 | *NRG4* | T | C | 0.025 | -0.094 | 5.40E-14 |
| rs12423664 | 12 | 133,069,894 | *FBRSL1* | A | G | 0.152 | 0.042 | 1.75E-13 |
| rs7315236 | 12 | 52,251,933 | *LOC105369971* | T | C | 0.357 | 0.029 | 1.91E-13 |
| rs10892354 | 11 | 119,238,381 | *USP2* | T | C | 0.380 | 0.030 | 2.60E-13 |
| rs198851 | 6 | 26,104,632 | *HIST1H4C* | T | G | 0.144 | 0.039 | 5.80E-13 |
| rs1440411 | 4 | 144,158,285 | *USP38* | T | C | 0.571 | -0.028 | 1.08E-12 |
| rs62286563 | 4 | 10,122,665 | *WDR1* | T | G | 0.979 | -0.103 | 1.16E-12 |
| rs11663816 | 18 | 57,876,227 | *MC4R* | T | C | 0.730 | -0.030 | 1.40E-12 |
| rs12987661 | 2 | 69,813,458 | *AAK1* | T | C | 0.866 | 0.041 | 1.44E-12 |
| rs10414501 | 19 | 50,259,674 | *TSKS* | C | G | 0.957 | -0.125 | 1.56E-12 |
| rs57070985 | 19 | 4,969,053 | *KDM4B* | A | G | 0.646 | 0.029 | 2.04E-12 |
| rs1800574 | 12 | 121,416,864 | *HNF1A* | T | C | 0.031 | -0.081 | 2.84E-12 |
| rs10480300 | 7 | 151,406,005 | *PRKAG2* | T | C | 0.276 | 0.030 | 4.26E-12 |
| rs455213 | 5 | 34,660,235 | *RAI14* | T | C | 0.543 | -0.027 | 6.05E-12 |
| rs2868194 | 19 | 33,350,060 | *SLC7A9* | T | C | 0.408 | -0.027 | 8.90E-12 |
| rs2867112 | 2 | 651,349 | *TMEM18* | T | G | 0.830 | 0.035 | 9.84E-12 |
| rs9420446 | 10 | 88,880,689 | *FAM35A* | T | C | 0.137 | -0.038 | 1.13E-11 |
| rs56106601 | 9 | 130,770,484 | *FAM102A* | A | C | 0.946 | 0.061 | 2.68E-11 |
| rs626277 | 13 | 72,347,696 | *DACH1* | A | C | 0.594 | 0.026 | 2.69E-11 |
| rs187355703 | 2 | 176,993,583 | *HOXD8* | C | G | 0.975 | -0.086 | 2.70E-11 |
| rs2760215 | 1 | 163,675,883 | *LOC100422212* | T | C | 0.503 | -0.025 | 5.81E-11 |
| rs4788815 | 16 | 71,634,811 | *TAT* | A | T | 0.357 | -0.026 | 7.44E-11 |
| rs742493 | 6 | 40,998,167 | *UNC5CL* | T | C | 0.881 | 0.039 | 9.79E-11 |
| rs1800961 | 20 | 43,042,364 | *HNF4A* | T | C | 0.034 | -0.076 | 1.63E-10 |
| rs8050136 | 16 | 53,816,275 | *FTO* | A | C | 0.403 | 0.025 | 2.34E-10 |
| rs12485100 | 22 | 44,325,516 | *PNPLA3* | T | G | 0.173 | -0.033 | 2.44E-10 |
| rs4997081 | 16 | 20,365,234 | *UMOD* | C | G | 0.196 | -0.030 | 4.18E-10 |
| rs1478604 | 15 | 39,873,321 | *THBS1* | T | C | 0.706 | -0.026 | 4.49E-10 |
| rs188917216 | 4 | 88,872,920 | *SPP1* | A | C | 0.988 | -0.143 | 6.44E-10 |
| rs2453580 | 17 | 19,438,321 | *SLC47A1* | T | C | 0.598 | 0.025 | 7.01E-10 |
| rs7303595 | 12 | 15,359,063 | *RERG* | A | T | 0.336 | 0.025 | 7.05E-10 |
| rs7640441 | 3 | 125,118,082 | *ZNF148* | A | C | 0.246 | -0.028 | 1.26E-09 |
| rs80120242 | 3 | 132,235,344 | *DNAJC13* | A | T | 0.947 | -0.062 | 1.87E-09 |
| rs861536 | 14 | 104,167,564 | *KLC1* | A | G | 0.621 | 0.024 | 2.16E-09 |
| rs74440730 | 10 | 16,920,892 | *CUBN* | A | C | 0.892 | -0.037 | 2.22E-09 |
| rs7267595 | 20 | 10,643,850 | *JAG1* | A | C | 0.510 | 0.023 | 3.15E-09 |
| rs6119510 | 20 | 33,287,782 | *TP53INP2* | T | G | 0.596 | -0.023 | 3.20E-09 |
| rs9288447 | 2 | 213,083,638 | *ERBB4* | T | C | 0.546 | -0.023 | 3.27E-09 |
| rs2929508 | 15 | 72,246,964 | *MYO9A* | A | T | 0.261 | -0.029 | 3.65E-09 |
| rs35198068 | 10 | 114,754,784 | *TCF7L2* | T | C | 0.706 | 0.025 | 5.85E-09 |
| rs8040109 | 15 | 73,334,225 | *NEO1* | A | C | 0.707 | 0.025 | 5.85E-09 |
| rs11718633 | 3 | 126,012,421 | *KLF15* | T | C | 0.198 | -0.028 | 7.05E-09 |
| rs1234413 | 2 | 148,844,369 | *MBD5* | T | C | 0.442 | -0.022 | 7.08E-09 |
| rs4646068 | 1 | 15,828,704 | *CASP9* | T | C | 0.692 | 0.024 | 7.71E-09 |
| rs759219 | 2 | 71,163,225 | *ATP6V1B1* | T | C | 0.439 | -0.022 | 7.93E-09 |
| rs72782806 | 2 | 15,788,511 | *DDX1* | A | G | 0.260 | 0.025 | 8.12E-09 |
| rs55781567 | 15 | 78,857,986 | *CHRNA5* | C | G | 0.655 | 0.023 | 1.11E-08 |
| rs11683692 | 2 | 145,509,615 | *TEX41* | T | C | 0.944 | -0.048 | 1.32E-08 |
| rs4808762 | 19 | 18,326,222 | *PDE4C* | T | C | 0.720 | -0.024 | 1.36E-08 |
| rs11644696 | 16 | 81,572,093 | *CMIP* | A | G | 0.477 | 0.022 | 1.41E-08 |
| rs35506085 | 11 | 2,165,576 | *IGF2* | A | G | 0.189 | -0.029 | 1.50E-08 |
| rs2472297 | 15 | 75,027,880 | *CYP1A1* | T | C | 0.249 | -0.028 | 1.50E-08 |
| rs219781 | 21 | 37,832,621 | *CLDN14* | T | G | 0.246 | -0.025 | 1.56E-08 |
| rs141990161 | 1 | 119,943,525 | *HAO2* | T | C | 0.985 | 0.133 | 1.59E-08 |
| rs7986094 | 13 | 31,029,931 | *HMGB1* | A | C | 0.302 | -0.024 | 1.74E-08 |
| rs2466077 | 8 | 32,432,753 | *NRG1* | T | G | 0.533 | -0.022 | 1.78E-08 |
| rs10956924 | 8 | 95,678,312 | *ESRP1* | T | C | 0.279 | -0.024 | 1.79E-08 |
| rs12472381 | 2 | 59,321,225 | *LINC01122* | A | G | 0.390 | 0.022 | 1.80E-08 |
| rs1047891 | 2 | 211,540,507 | *CPS1* | A | C | 0.311 | -0.024 | 2.09E-08 |
| rs10857147 | 4 | 81,181,072 | *FGF5* | A | T | 0.713 | 0.024 | 2.21E-08 |
| rs35396326 | 19 | 45,357,003 | *NECTIN2* | C | G | 0.704 | 0.025 | 2.31E-08 |
| rs11551890 | 7 | 97,845,713 | *TECPR1* | A | G | 0.509 | 0.023 | 2.40E-08 |
| rs76004499 | 5 | 176,705,865 | *NSD1* | C | G | 0.972 | -0.074 | 3.27E-08 |
| rs12037861 | 1 | 221,038,177 | *HLX-AS1* | A | T | 0.704 | 0.023 | 3.39E-08 |
| rs98270 | 4 | 48,019,323 | *NIPAL1* | A | G | 0.362 | 0.022 | 4.21E-08 |
| rs11128111 | 3 | 69,145,632 | *ARL6IP5* | T | C | 0.480 | -0.021 | 4.64E-08 |
| rs139428292 | 1 | 145,507,646 | *NBPF20* | A | G | 0.027 | -0.073 | 4.91E-08 |
| rs62294340 | 3 | 169,155,476 | *MECOM* | A | G | 0.364 | -0.022 | 5.00E-08 |
| rs148185902 | 11 | 30,718,534 | *MPPED2* | A | G | 0.012 | 0.123 | 5.76E-08 |
| Four SNVs that were not included due to poor imputation quality | | | | | | | | |
| rs142773928 | 20 | 43,038,720 | *HNF4A* | A | G | 0.166 | 0.032 | 3.77E-09 |
| rs2480712 | 1 | 2,156,999 | *SKI* | C | G | 0.662 | 0.024 | 6.25E-09 |
| rs10942549 | 5 | 72,426,137 | *TMEM171* | C | G | 0.312 | -0.042 | 1.64E-22 |
| rs10405423 | 19 | 7,211,311 | *INSR* | A | C | 0.663 | 0.039 | 1.07E-20 |

Table was adopted from the supplementary table 18 from the referenced consortium GWAS meta-analysis (Tin et al., 2019). SNV: single nucleotide variant; Chr: chromosome; EA: effect allele; OA: other allele; EAF: effect allele frequency.

## Supplementary table 2. SNPs derived from trans-ethnic meta-GWAS used to calculate the urate-PRS

| SNV | Chr | Position(b37) | Nearest gene | EA | OA | EAF | Weight | p-value |
| --- | --- | --- | --- | --- | --- | --- | --- | --- |
| rs3775947 | 4 | 9995240 | *SLC2A9* | T | C | 0.69 | 0.277 | 0.00E+00 |
| rs74904971 | 4 | 89050026 | *ABCG2* | A | C | 0.20 | 0.217 | 0.00E+00 |
| rs1359232 | 6 | 25809716 | *SLC17A1* | A | C | 0.40 | -0.089 | 5.59E-159 |
| rs12504795 | 4 | 10499344 | *CLNK* | T | C | 0.74 | 0.075 | 4.25E-101 |
| rs531763 | 11 | 64352063 | *SLC22A12* | A | G | 0.56 | -0.116 | 1.58E-246 |
| rs1260326 | 2 | 27730940 | *GCKR* | T | C | 0.44 | 0.066 | 2.97E-95 |
| rs1171617 | 10 | 61467182 | *SLC16A9* | T | G | 0.77 | 0.081 | 2.14E-77 |
| rs73119306 | 12 | 57826982 | *INHBC* | A | G | 0.79 | 0.071 | 7.45E-65 |
| rs10910845 | 1 | 145723120 | *NBPF20/PDZK1** | A | C | 0.54 | 0.060 | 1.10E-69 |
| rs3904600 | 6 | 7109665 | *RREB1* | C | G | 0.40 | 0.057 | 5.23E-51 |
| rs2070803 | 1 | 155157715 | *TRIM46* | A | G | 0.50 | 0.054 | 2.12E-60 |
| rs10994860 | 10 | 52645424 | *A1CF* | T | C | 0.17 | 0.061 | 7.44E-42 |
| rs2244552 | 3 | 53055522 | *SFMBT1* | A | G | 0.55 | -0.043 | 1.06E-43 |
| rs4014195 | 11 | 65506822 | *RNASEH2C* | C | G | 0.69 | -0.051 | 6.87E-52 |
| rs12908437 | 15 | 99287375 | *IGF1R* | T | C | 0.43 | 0.045 | 3.77E-46 |
| rs11072567 | 15 | 76298744 | *NRG4* | A | G | 0.50 | -0.042 | 2.52E-39 |
| rs10223666 | 6 | 43805502 | *VEGFA* | C | G | 0.73 | 0.044 | 1.27E-32 |
| rs2941487 | 8 | 76480350 | *HNF4G* | T | C | 0.53 | -0.043 | 5.68E-44 |
| rs6820627 | 4 | 9491205 | *DEFB131A* | A | G | 0.07 | -0.100 | 4.66E-21 |
| rs1051921 | 7 | 73007943 | *MLXIPL* | A | G | 0.17 | -0.051 | 2.86E-34 |
| rs9895661 | 17 | 59456589 | *BCAS3* | T | C | 0.67 | 0.053 | 7.63E-45 |
| rs3794748 | 17 | 53365172 | *HLF* | A | G | 0.35 | 0.033 | 8.68E-23 |
| rs35942569 | 6 | 26339131 | *BTN3A2* | A | G | 0.91 | -0.061 | 1.19E-19 |
| rs34861762 | 8 | 23748420 | *STC1* | T | C | 0.37 | 0.034 | 1.62E-25 |
| rs1649078 | 10 | 60293320 | *BICC1* | A | C | 0.51 | -0.045 | 2.68E-39 |
| rs62052820 | 16 | 69575238 | *MIR1538* | A | G | 0.19 | 0.043 | 1.17E-26 |
| rs57652769 | 16 | 79753976 | *MAFTRR* | T | C | 0.30 | -0.037 | 2.32E-27 |
| rs2075251 | 2 | 170011458 | *LRP2* | A | T | 0.66 | -0.037 | 7.01E-27 |
| rs10774625 | 12 | 111910219 | *ATXN2* | A | G | 0.47 | 0.032 | 1.29E-17 |
| rs62435145 | 7 | 1286567 | *UNCX* | T | G | 0.53 | 0.040 | 3.80E-26 |
| rs148015593 | 12 | 122523668 | *MLXIP* | T | G | 0.50 | 0.029 | 3.42E-21 |
| rs62128132 | 19 | 50217955 | *CPT1C* | T | C | 0.97 | -0.115 | 1.99E-15 |
| rs17050272 | 2 | 121306440 | *LINC01101* | A | G | 0.43 | 0.028 | 1.01E-17 |
| rs62140395 | 2 | 28244926 | *BABAM2* | C | G | 0.12 | 0.050 | 8.68E-15 |
| rs699465 | 3 | 52310442 | *WDR82* | A | G | 0.15 | 0.033 | 4.60E-13 |
| rs79598313 | 1 | 27284913 | *KDF1* | T | C | 0.03 | 0.102 | 1.16E-15 |
| rs57440165 | 6 | 26843517 | *GUSBP2* | A | C | 0.92 | -0.060 | 4.25E-15 |
| rs4897160 | 6 | 126223944 | *NCOA7* | A | G | 0.47 | 0.024 | 2.04E-14 |
| rs11204682 | 1 | 150595537 | *ENSA* | T | G | 0.19 | 0.032 | 6.00E-14 |
| rs7868781 | 9 | 33141320 | *B4GALT1* | A | G | 0.61 | 0.030 | 5.34E-20 |
| rs963837 | 11 | 30749090 | *DCDC1* | T | C | 0.59 | 0.034 | 4.75E-25 |
| rs35501037 | 6 | 27739566 | *LOC100131289* | A | T | 0.09 | 0.052 | 3.36E-14 |
| rs7752448 | 6 | 28301099 | *ZSCAN31* | A | G | 0.88 | -0.040 | 7.44E-15 |
| rs11217257 | 11 | 119238455 | *USP2* | A | G | 0.67 | -0.027 | 2.81E-15 |
| rs74397112 | 12 | 133094111 | *FBRSL1* | T | C | 0.15 | 0.043 | 1.34E-14 |
| rs1440411 | 4 | 144158285 | *USP38* | T | C | 0.61 | -0.027 | 1.18E-15 |
| rs11663816 | 18 | 57876227 | *MC4R* | T | C | 0.75 | -0.026 | 1.25E-12 |
| rs12987661 | 2 | 69813458 | *AAK1* | T | C | 0.88 | 0.043 | 4.32E-16 |
| rs17696736 | 12 | 112486818 | *NAA25* | A | G | 0.58 | -0.028 | 2.43E-13 |
| rs429479 | 6 | 29372323 | *OR12D2* | A | G | 0.90 | -0.047 | 2.02E-12 |
| rs1800574 | 12 | 121416864 | *HNF1A* | T | C | 0.03 | -0.085 | 2.57E-14 |
| rs3118365 | 6 | 28839908 | *LINC01623* | A | G | 0.09 | 0.046 | 5.81E-12 |
| rs4807003 | 19 | 4957133 | *UHRF1* | A | G | 0.32 | -0.028 | 8.85E-12 |
| rs11169926 | 12 | 52248787 | *LOC105369971* | A | C | 0.34 | 0.030 | 4.43E-18 |
| rs455213 | 5 | 34660235 | *RAI14* | T | C | 0.60 | -0.023 | 2.07E-12 |
| rs73728279 | 7 | 151411494 | *PRKAG2* | T | G | 0.27 | 0.033 | 1.15E-14 |
| rs6746275 | 2 | 635857 | *TMEM18* | A | C | 0.85 | 0.035 | 5.50E-16 |
| rs9420446 | 10 | 88880689 | *FAM35A* | T | C | 0.31 | -0.041 | 1.37E-20 |
| rs77951490 | 6 | 25236645 | *LOC101928663* | A | G | 0.05 | 0.060 | 2.59E-12 |
| rs12368865 | 12 | 58422642 | *LINC02403* | A | G | 0.91 | 0.045 | 1.31E-11 |
| rs116183010 | 4 | 88468158 | *SPARCL1* | A | G | 0.02 | 0.096 | 3.66E-12 |
| rs626277 | 13 | 72347696 | *DACH1* | A | C | 0.50 | 0.025 | 3.78E-14 |
| rs2760215 | 1 | 163675883 | *LOC100422212* | T | C | 0.45 | -0.022 | 7.55E-12 |
| rs4788815 | 16 | 71634811 | *TAT* | A | T | 0.34 | -0.024 | 2.93E-13 |
| rs742493 | 6 | 40998167 | *UNC5CL* | T | C | 0.89 | 0.035 | 8.26E-11 |
| rs12484809 | 22 | 44325631 | *PNPLA3* | T | C | 0.28 | -0.035 | 1.73E-20 |
| rs10418164 | 19 | 33411139 | *CEP89* | T | G | 0.45 | 0.025 | 3.14E-15 |
| rs10198459 | 2 | 177273272 | *MTX2* | T | C | 0.25 | 0.027 | 1.27E-13 |
| rs62585312 | 9 | 130723419 | *FAM102A* | C | G | 0.93 | 0.053 | 4.11E-15 |
| rs62517932 | 8 | 77031593 | *LINC01111* | A | G | 0.08 | 0.041 | 3.04E-09 |
| rs2453580 | 17 | 19438321 | *SLC47A1* | T | C | 0.64 | 0.022 | 3.49E-10 |
| rs62033406 | 16 | 53824226 | *FTO* | A | G | 0.55 | -0.019 | 3.23E-09 |
| rs11070231 | 15 | 40021576 | *FSIP1* | A | C | 0.57 | -0.028 | 2.07E-17 |
| rs861536 | 14 | 104167564 | *KLC1* | A | G | 0.67 | 0.021 | 2.81E-09 |
| rs78946096 | 3 | 132188163 | *DNAJC13* | A | G | 0.95 | -0.060 | 1.48E-09 |
| rs74440730 | 10 | 16920892 | *CUBN* | A | C | 0.89 | -0.039 | 5.50E-11 |
| rs2957740 | 15 | 72307691 | *MYO9A* | A | G | 0.41 | -0.024 | 3.38E-11 |
| rs9859616 | 3 | 125149488 | *SNX4* | A | G | 0.22 | -0.024 | 4.17E-10 |
| rs35198068 | 10 | 114754784 | *TCF7L2* | T | C | 0.72 | 0.028 | 2.57E-13 |
| rs11718633 | 3 | 126012421 | *KLF15* | T | C | 0.19 | -0.027 | 7.64E-09 |
| rs1949651 | 2 | 213117629 | *ERBB4* | T | C | 0.52 | 0.021 | 2.64E-11 |
| rs6119524 | 20 | 33373813 | *NCOA6* | T | C | 0.34 | 0.020 | 3.50E-09 |
| rs759219 | 2 | 71163225 | *ATP6V1B1* | T | C | 0.41 | -0.018 | 9.23E-09 |
| rs9302635 | 16 | 72144174 | *DHX38* | T | C | 0.76 | -0.028 | 2.24E-09 |
| rs72782806 | 2 | 15788511 | *DDX1* | A | G | 0.23 | 0.023 | 1.02E-09 |
| rs494268 | 15 | 76815713 | *SCAPER* | T | C | 0.91 | 0.032 | 3.96E-09 |
| rs12644329 | 4 | 143634746 | *INPP4B* | A | G | 0.64 | -0.019 | 8.47E-09 |
| rs10803394 | 1 | 15909480 | *AGMAT* | C | G | 0.37 | -0.020 | 5.83E-10 |
| rs6031598 | 20 | 43056149 | *HNF4A* | T | G | 0.54 | -0.022 | 1.73E-12 |
| rs2307394 | 2 | 148716428 | *ORC4* | T | C | 0.63 | -0.020 | 1.05E-09 |
| rs11683692 | 2 | 145509615 | *TEX41* | T | C | 0.95 | -0.047 | 1.55E-08 |
| rs11644696 | 16 | 81572093 | *CMIP* | A | G | 0.41 | 0.019 | 6.42E-09 |
| rs2472297 | 15 | 75027880 | *CYP1A1* | T | C | 0.24 | -0.028 | 8.49E-09 |
| rs219781 | 21 | 37832621 | *CLDN14* | T | G | 0.25 | -0.027 | 2.22E-10 |
| rs141990161 | 1 | 119943525 | *HAO2* | T | C | 0.98 | 0.133 | 1.59E-08 |
| rs9579574 | 13 | 31025634 | *HMGB1* | A | G | 0.29 | -0.020 | 2.03E-08 |
| rs1047891 | 2 | 211540507 | *CPS1* | A | C | 0.29 | -0.024 | 1.91E-11 |
| rs10857147 | 4 | 81181072 | *FGF5* | A | T | 0.71 | 0.028 | 1.67E-15 |
| rs35232147 | 4 | 89916224 | *FAM13A* | T | C | 0.37 | 0.018 | 3.37E-08 |
| rs11551890 | 7 | 97845713 | *TECPR1* | A | G | 0.49 | 0.024 | 2.05E-09 |
| rs7005606 | 8 | 32401501 | *NRG1* | T | G | 0.60 | -0.021 | 9.56E-11 |
| rs12625256 | 20 | 10638386 | *JAG1* | A | T | 0.59 | 0.019 | 1.93E-09 |
| rs6730325 | 2 | 59315828 | *LINC01122* | A | G | 0.67 | -0.019 | 2.58E-08 |
| rs8039418 | 15 | 73441432 | *NEO1* | T | C | 0.47 | -0.018 | 2.39E-08 |
| rs62294340 | 3 | 169155476 | *MECOM* | A | G | 0.35 | -0.021 | 7.62E-11 |
| rs77924615 | 16 | 20392332 | *PDILT* | A | G | 0.20 | -0.027 | 1.27E-11 |
| rs164011 | 17 | 74273165 | *QRICH2* | A | G | 0.41 | -0.021 | 1.05E-10 |
| rs1533096 | 4 | 4784139 | *LOC101928279* | C | G | 0.59 | 0.020 | 3.16E-08 |
| rs12496412 | 3 | 141741823 | *TFDP2* | A | G | 0.67 | 0.021 | 5.30E-10 |
| rs3741210 | 11 | 2169540 | *IGF2* | A | G | 0.66 | 0.019 | 1.17E-08 |
| rs7572603 | 2 | 61552145 | *USP34* | C | G | 0.64 | -0.023 | 3.62E-12 |
| rs7039 | 3 | 69154343 | *ARL6IP5* | C | G | 0.45 | -0.021 | 5.24E-11 |
| rs12548367 | 8 | 95929202 | *TP53INP1* | T | C | 0.68 | 0.024 | 4.39E-13 |
| rs56338130 | 19 | 18235871 | *MAST3* | T | C | 0.22 | 0.023 | 4.15E-09 |
| rs2356864 | 1 | 50839740 | *DMRTA2* | A | G | 0.57 | 0.020 | 1.69E-09 |
| rs11097693 | 4 | 101121391 | *LOC101929353* | A | G | 0.58 | -0.019 | 9.03E-10 |
| rs1407040 | 20 | 57472174 | *GNAS* | T | C | 0.69 | -0.025 | 5.70E-14 |
| rs753725 | 6 | 30890871 | *VARS2* | T | C | 0.41 | 0.021 | 1.05E-09 |
| rs56129505 | 1 | 186718261 | *PACERR* | T | C | 0.29 | 0.021 | 2.61E-08 |
| rs116379131 | 5 | 39961618 | *LINC00603* | A | T | 0.03 | -0.058 | 3.15E-08 |
| rs7212936 | 17 | 1646651 | *SERPINF2* | A | C | 0.45 | -0.018 | 1.75E-08 |
| rs6805417 | 3 | 142762487 | *U2SURP* | T | C | 0.34 | 0.019 | 1.10E-08 |
| rs11781985 | 8 | 8589783 | *CLDN23* | T | C | 0.80 | -0.025 | 7.38E-10 |
| rs6707386 | 2 | 113981022 | *PAX8* | A | G | 0.35 | 0.018 | 3.93E-08 |
| rs35229181 | 11 | 30241698 | *FSHB* | A | G | 0.79 | 0.022 | 5.56E-09 |
| rs10177191 | 2 | 9246721 | *ASAP2* | T | C | 0.40 | -0.018 | 3.05E-08 |
| rs11066390 | 12 | 113163766 | *RPH3A* | A | G | 0.27 | 0.025 | 2.97E-11 |
| rs2970581 | 1 | 212083512 | *INTS7* | A | G | 0.04 | 0.059 | 3.78E-09 |
| rs2219647 | 16 | 51733405 | *LINC01571* | A | G | 0.27 | 0.021 | 5.38E-09 |
| rs584425 | 1 | 48536798 | *SKINT1L* | A | G | 0.33 | -0.021 | 1.36E-10 |
| rs1457231 | 2 | 161101562 | *LINC02478* | T | C | 0.70 | 0.019 | 3.17E-08 |
| rs8022225 | 14 | 55767069 | *FBXO34* | A | G | 0.56 | -0.018 | 1.81E-08 |
| rs9415676 | 10 | 65010626 | *JMJD1C* | A | G | 0.65 | -0.019 | 4.53E-09 |
| rs10438961 | 18 | 42779107 | *SLC14A2* | T | G | 0.73 | -0.021 | 2.01E-08 |
| rs6138584 | 20 | 25463148 | *NINL* | A | T | 0.24 | 0.023 | 2.50E-09 |
| rs836968 | 12 | 50267335 | *FAIM2* | T | C | 0.37 | -0.020 | 4.56E-09 |
| rs3769810 | 2 | 183037246 | *PDE1A* | A | G | 0.76 | 0.022 | 2.81E-09 |
| rs2834319 | 21 | 35357025 | *LOC101928126* | T | C | 0.85 | -0.026 | 8.90E-09 |
| rs2436962 | 8 | 103645813 | *KLF10* | A | G | 0.18 | 0.026 | 2.51E-10 |
| rs73058028 | 3 | 31528128 | *STT3B* | C | G | 0.64 | 0.018 | 1.54E-08 |
| rs10886117 | 10 | 119480578 | *EMX2* | A | G | 0.26 | 0.028 | 2.42E-12 |
| rs8024386 | 15 | 90670526 | *IDH2* | A | C | 0.77 | -0.022 | 2.12E-08 |
| rs10890263 | 1 | 44061032 | *PTPRF* | T | C | 0.74 | -0.020 | 4.83E-08 |
| rs72804857 | 2 | 27161476 | *DPYSL5* | C | G | 0.15 | 0.028 | 2.53E-10 |
| rs4073582 | 11 | 66050712 | *CNIH2* | A | G | 0.31 | -0.041 | 5.41E-28 |
| rs662026 | 1 | 91531022 | *ZNF644* | A | G | 0.80 | 0.021 | 3.26E-08 |
| rs73611258 | 20 | 39742284 | *TOP1* | A | G | 0.65 | 0.024 | 4.03E-10 |
| rs7259484 | 19 | 1813207 | *ATP8B3* | A | G | 0.65 | 0.021 | 3.60E-09 |
| rs3174352 | 9 | 95219248 | *CENPP* | A | G | 0.46 | -0.019 | 3.41E-09 |
| rs1800977 | 9 | 107690450 | *ABCA1* | A | G | 0.32 | -0.019 | 7.95E-09 |
| rs174594 | 11 | 61619829 | *FADS2* | A | C | 0.61 | -0.018 | 4.88E-08 |
| rs60388273 | 3 | 149214543 | *TM4SF4* | A | G | 0.23 | 0.030 | 3.15E-11 |
| rs10901057 | 9 | 134245698 | *PRRC2B* | C | G | 0.26 | -0.026 | 2.71E-08 |
| rs4149056 | 12 | 21331549 | *SLCO1B1* | T | C | 0.85 | 0.024 | 2.22E-08 |
| rs7209801 | 17 | 42323376 | *SLC4A1* | A | G | 0.40 | 0.019 | 5.19E-09 |
| rs28362590 | 5 | 176731452 | *PRELID1* | T | G | 0.66 | 0.021 | 1.05E-09 |
| rs9271585 | 6 | 32590879 | *HLA-DQA1* | A | C | 0.33 | -0.025 | 6.94E-12 |
| rs2058787 | 12 | 15208908 | *LINC01489* | A | G | 0.32 | -0.020 | 5.75E-09 |
| rs3850445 | 9 | 16703381 | *BNC2* | A | G | 0.38 | 0.021 | 1.69E-08 |
| rs2645477 | 17 | 57845624 | *VMP1* | A | C | 0.52 | 0.018 | 7.66E-09 |
| rs142874192 | 2 | 54900899 | *SPTBN1* | C | G | 0.96 | -0.059 | 1.08E-08 |
| rs113704612 | 2 | 158294018 | *CYTIP* | T | G | 0.04 | -0.080 | 1.14E-11 |
| rs7110302 | 11 | 66690454 | *PC* | T | C | 0.38 | -0.018 | 4.56E-08 |
| rs12669187 | 7 | 30915478 | *INMT-MINDY4* | A | G | 0.14 | 0.032 | 1.25E-08 |
| rs56230350 | 16 | 68166971 | *NFATC3* | A | C | 0.87 | 0.026 | 3.08E-08 |
| rs11940694 | 4 | 39414993 | *KLB* | A | G | 0.46 | -0.017 | 2.37E-08 |
| rs10084334 | 2 | 37250891 | *HEATR5B* | C | G | 0.62 | 0.019 | 4.33E-08 |
| rs9373896 | 6 | 107169822 | *LOC100422737* | A | T | 0.15 | 0.030 | 9.09E-09 |
| rs143825439 | 11 | 63319993 | *HRASLS2* | T | G | 0.05 | -0.224 | 1.23E-76 |
| rs34888828 | 11 | 64968104 | *CAPN1* | A | G | 0.11 | -0.057 | 1.45E-23 |
| rs148838714 | 11 | 62732352 | *SLC22A6* | A | G | 0.07 | -0.074 | 2.14E-16 |
| rs17550549 | 12 | 111357471 | *MYL2* | T | C | 0.14 | -0.035 | 5.18E-11 |
| Eleven SNVs that were not included due to poor imputation quality | | | | | | | | |
| rs10942549 | 5 | 72426137 | *TMEM171* | C | G | 0.30 | -0.040 | 1.09E-30 |
| rs10405423 | 19 | 7211311 | *INSR* | A | C | 0.70 | 0.033 | 1.11E-18 |
| rs12134456 | 1 | 155722506 | *GON4L* | C | G | 0.63 | -0.044 | 6.33E-20 |
| rs56401710 | 6 | 43269180 | *SLC22A7* | A | C | 0.59 | -0.042 | 5.00E-30 |
| rs2304667 | 2 | 121989489 | *TFCP2L1* | A | G | 0.42 | 0.023 | 1.02E-09 |
| rs73436803 | 15 | 75619201 | *COMMD4* | T | C | 0.24 | -0.027 | 2.58E-12 |
| rs4962699 | 10 | 126477209 | *EEF1AKMT2* | A | G | 0.25 | 0.020 | 1.65E-08 |
| rs1006207 | 11 | 63849812 | *MACROD1* | T | C | 0.56 | -0.047 | 3.54E-41 |
| rs7757144 | 6 | 1997865 | *GMDS* | A | G | 0.63 | -0.022 | 2.31E-11 |
| rs7417952 | 1 | 93854186 | *DR1* | C | G | 0.40 | -0.023 | 2.75E-10 |
| rs11227805 | 11 | 67246757 | *AIP* | T | C | 0.18 | -0.027 | 8.72E-09 |

Table was adopted from the supplementary table 3 from the referenced consortium GWAS meta-analysis (Tin et al., 2019). SNV: single nucleotide variant; Chr: chromosome; EA: effect allele; OA: other allele; EAF: effect allele frequency.

## Supplementary table 3. Established PRS for other traits and diseases extracted from PGS database

| PGSID | Reported  Trait | PGS Method | Genome Build | # Variants | # used | % | PMID | FTP link |
| --- | --- | --- | --- | --- | --- | --- | --- | --- |
| PGS000115 | LDL | Established  lipid loci | GRCh37 | 223 | 171 | 76.7 | 32049305 | http://ftp.ebi.ac.uk/pub/databases/  spot/pgs/scores/PGS000115/  ScoringFiles/PGS000115.txt.gz |
| PGS000027 | Body Mass  Index | LDpred | hg19 | 2100302 | 1887368 | 89.9 | 31002795 | http://ftp.ebi.ac.uk/pub/databases/  spot/pgs/scores/PGS000027/  ScoringFiles/PGS000027.txt.gz |
| PGS000330 | Type 2  diabetes | LDpred | hg19 | 6437380 | 5060532 | 78.6 | 32273609 | http://ftp.ebi.ac.uk/pub/databases/  spot/pgs/scores/PGS000330/  ScoringFiles/PGS000330.txt.gz |
| PGS000331 | Atrial  fibrillation | LDpred | hg19 | 6183494 | 4686237 | 75.8 | 32273609 | http://ftp.ebi.ac.uk/pub/databases/  spot/pgs/scores/PGS000331/  ScoringFiles/PGS000331.txt.gz |
| PGS000332 | Breast  cancer | LDpred | hg19 | 6390808 | 4920524 | 77.0 | 32273609 | http://ftp.ebi.ac.uk/pub/databases/  spot/pgs/scores/PGS000332/  ScoringFiles/PGS000332.txt.gz |
| PGS000333 | Prostate  cancer | LDpred | hg19 | 6606785 | 5032578 | 76.2 | 32273609 | http://ftp.ebi.ac.uk/pub/databases/  spot/pgs/scores/PGS000333/  ScoringFiles/PGS000333.txt.gz |
| PGS000329 | Coronary  heart  disease | LDpred | hg19 | 6423165 | 5063922 | 78.8 | 32273609 | http://ftp.ebi.ac.uk/pub/databases/  spot/pgs/scores/PGS000329/  ScoringFiles/PGS000329.txt.gz |
| PGS000194 | Rheumatoid  Arthritis (CCP-positive) | P+T | GRCh38 | 209* | 154 | 73.7 | 32461333 | http://ftp.ebi.ac.uk/pub/databases/  spot/pgs/scores/PGS000194/  ScoringFiles/PGS000194.txt.gz |
| PGS000197 | Spondylo-  arthropathy | P+T | GRCh38 | 209* | 154 | 73.7 | 32461333 | http://ftp.ebi.ac.uk/pub/databases/  spot/pgs/scores/PGS000197/  ScoringFiles/PGS000197.txt.gz |
| PGS000198 | Psoriatic  arthritis | P+T | GRCh38 | 209* | 154 | 73.7 | 32461333 | http://ftp.ebi.ac.uk/pub/databases/  spot/pgs/scores/PGS000198/  ScoringFiles/PGS000198.txt.gz |

PGS: polygenic score; LDL: low density lipoprotein cholesterol; P+T: Pruning and Thresholding; *: number after removing variants in HLA region.

## Supplementary table 4. Phenotypes with raw p-value <0.05 in urate-PRS PheWAS

| Phecode | beta | L95 | U95 | p-value | Case N | Ctrl. N | Phenotype name | Category |
| --- | --- | --- | --- | --- | --- | --- | --- | --- |
| 274.1 | **1.495** | **1.372** | **1.619** | **4.37E-124** | **2858** | **41133** | **Gout** | **endocrine/metabolic** |
| 274 | **1.453** | **1.331** | **1.575** | **3.96E-120** | **2929** | **41133** | **Gout and other crystal arthropathies** | **endocrine/metabolic** |
| 274.11 | **1.664** | **1.454** | **1.875** | **3.24E-54** | **912** | **41133** | **Gouty arthropathy** | **endocrine/metabolic** |
| 401.1 | **0.188** | **0.116** | **0.260** | **3.31E-07** | **26346** | **15984** | **Essential hypertension** | **circulatory system** |
| 401 | **0.185** | **0.113** | **0.257** | **4.66E-07** | **26556** | **15984** | **Hypertension** | **circulatory system** |
| 585.33 | **0.207** | **0.110** | **0.304** | **2.88E-05** | **5612** | **33360** | **Chronic Kidney Disease, Stage III** | **genitourinary** |
| 585.34 | **0.429** | **0.226** | **0.631** | **3.36E-05** | **972** | **33360** | **Chronic Kidney Disease, Stage IV** | **genitourinary** |
| 440 | 0.279 | 0.139 | 0.420 | 9.67E-05 | 2160 | 35748 | Atherosclerosis | circulatory system |
| 401.2 | 0.280 | 0.137 | 0.423 | 1.21E-04 | 3571 | 15984 | Hypertensive heart and/or renal disease | circulatory system |
| 401.22 | 0.391 | 0.191 | 0.590 | 1.21E-04 | 1427 | 15984 | Hypertensive chronic kidney disease | circulatory system |
| 585.3 | 0.180 | 0.087 | 0.272 | 1.41E-04 | 6207 | 33360 | Chronic renal failure [CKD] | genitourinary |
| 585 | 0.165 | 0.079 | 0.252 | 1.81E-04 | 7219 | 33360 | Renal failure | genitourinary |
| 411.2 | 0.207 | 0.092 | 0.322 | 4.20E-04 | 3477 | 32289 | Myocardial infarction | circulatory system |
| 440.2 | 0.271 | 0.108 | 0.434 | 1.14E-03 | 1540 | 35748 | Atherosclerosis of the extremities | circulatory system |
| 732 | 1.084 | 0.406 | 1.761 | 1.71E-03 | 83 | 36642 | Osteochondropathies | musculoskeletal |
| 272.13 | 0.176 | 0.066 | 0.286 | 1.73E-03 | 6035 | 12299 | Mixed hyperlipidemia | endocrine/metabolic |
| 669 | -1.016 | -1.655 | -0.376 | 1.86E-03 | 98 | 25584 | Complications of labor and delivery NEC | pregnancy complications |
| 502 | 0.558 | 0.205 | 0.911 | 1.94E-03 | 301 | 38694 | Postinflammatory pulmonary fibrosis | respiratory |
| 272.11 | 0.136 | 0.050 | 0.223 | 1.96E-03 | 16825 | 12299 | Hypercholesterolemia | endocrine/metabolic |
| 442 | 0.226 | 0.079 | 0.372 | 2.53E-03 | 1942 | 35748 | Other aneurysm | circulatory system |
| 585.4 | 0.484 | 0.166 | 0.801 | 2.85E-03 | 375 | 33360 | Chronic kidney disease, Stage I or II | genitourinary |
| 305.2 | -0.414 | -0.696 | -0.133 | 3.93E-03 | 549 | 19048 | Eating disorder | mental disorders |
| 202.21 | 1.194 | 0.373 | 2.016 | 4.37E-03 | 55 | 43470 | Nodular lymphoma | neoplasms |
| 276.5 | -0.291 | -0.492 | -0.089 | 4.62E-03 | 955 | 32033 | Hypovolemia | endocrine/metabolic |
| 586.2 | 0.339 | 0.104 | 0.574 | 4.75E-03 | 699 | 33360 | Cyst of kidney, acquired | genitourinary |
| 272 | 0.107 | 0.033 | 0.181 | 4.84E-03 | 29929 | 12299 | Disorders of lipoid metabolism | endocrine/metabolic |
| 250.21 | 0.763 | 0.231 | 1.295 | 4.92E-03 | 135 | 27005 | Type 2 diabetes with ketoacidosis | endocrine/metabolic |
| 272.1 | 0.106 | 0.032 | 0.181 | 5.04E-03 | 29898 | 12299 | Hyperlipidemia | endocrine/metabolic |
| 720 | -0.160 | -0.274 | -0.047 | 5.42E-03 | 3424 | 29987 | Spinal stenosis | musculoskeletal |
| 626.15 | 0.822 | 0.241 | 1.403 | 5.58E-03 | 120 | 9030 | Infertility, female, associated with anovulation | genitourinary |
| 571.6 | 1.212 | 0.353 | 2.071 | 5.71E-03 | 50 | 34148 | Primary biliary cirrhosis | digestive |
| 720.1 | -0.179 | -0.306 | -0.051 | 5.98E-03 | 2650 | 29987 | Spinal stenosis of lumbar region | musculoskeletal |
| 726.3 | 0.232 | 0.066 | 0.397 | 6.00E-03 | 1457 | 22266 | Bursitis | musculoskeletal |
| 876 | 1.057 | 0.303 | 1.811 | 6.03E-03 | 66 | 34273 | Posttraumatic wound infection not elsewhere classified | injuries & poisonings |
| 454 | -0.210 | -0.364 | -0.055 | 7.84E-03 | 1680 | 28627 | Varicose veins | circulatory system |
| 643.1 | -0.907 | -1.575 | -0.238 | 7.85E-03 | 88 | 25579 | Hyperemesis gravidarum | pregnancy complications |
| 411.8 | 0.146 | 0.037 | 0.255 | 8.78E-03 | 4028 | 32289 | Other chronic ischemic heart disease, unspecified | circulatory system |
| 401.21 | 0.223 | 0.056 | 0.390 | 8.84E-03 | 2261 | 15984 | Hypertensive heart disease | circulatory system |
| 695.7 | 0.340 | 0.085 | 0.595 | 8.88E-03 | 583 | 34329 | Prurigo and Lichen | dermatologic |
| 608 | -0.391 | -0.684 | -0.097 | 9.09E-03 | 462 | 8103 | Other disorders of male genital organs | genitourinary |
| 411 | 0.101 | 0.024 | 0.177 | 9.79E-03 | 10119 | 32289 | Ischemic Heart Disease | circulatory system |
| 440.22 | 0.257 | 0.061 | 0.453 | 1.01E-02 | 1035 | 35748 | Atherosclerosis of native arteries of the extremities with intermittent claudication | circulatory system |
| 513.31 | -0.932 | -1.645 | -0.219 | 1.04E-02 | 74 | 42478 | Apnea | respiratory |
| 588 | 0.347 | 0.081 | 0.612 | 1.05E-02 | 546 | 33360 | Disorders resulting from impaired renal function | genitourinary |
| 854 | 0.418 | 0.097 | 0.738 | 1.06E-02 | 370 | 40425 | Complications of cardiac/vascular device, implant, and graft | injuries & poisonings |
| 433.1 | 0.153 | 0.035 | 0.270 | 1.07E-02 | 3206 | 37400 | Occlusion and stenosis of precerebral arteries | circulatory system |
| 626.13 | 0.214 | 0.049 | 0.378 | 1.08E-02 | 1982 | 9030 | Irregular menstrual cycle | genitourinary |
| 255.11 | 1.106 | 0.248 | 1.964 | 1.15E-02 | 52 | 38173 | Cushing's syndrome | endocrine/metabolic |
| 512.7 | 0.127 | 0.028 | 0.225 | 1.20E-02 | 5000 | 20191 | Shortness of breath | respiratory |
| 585.2 | 0.333 | 0.072 | 0.593 | 1.25E-02 | 572 | 33360 | Renal failure NOS | genitourinary |
| 790.6 | 0.107 | 0.022 | 0.192 | 1.37E-02 | 6144 | 35072 | Other abnormal blood chemistry | symptoms |
| 272.12 | 0.209 | 0.042 | 0.377 | 1.42E-02 | 1637 | 12299 | Hyperglyceridemia | endocrine/metabolic |
| 285.2 | 0.237 | 0.046 | 0.428 | 1.50E-02 | 1095 | 28619 | Anemia of chronic disease | hematopoietic |
| 361 | -0.302 | -0.548 | -0.056 | 1.60E-02 | 633 | 35664 | Retinal detachments and defects | sense organs |
| 214.1 | -0.418 | -0.761 | -0.075 | 1.70E-02 | 320 | 42389 | Lipoma of skin and subcutaneous tissue | neoplasms |
| 586 | 0.167 | 0.030 | 0.305 | 1.73E-02 | 2173 | 33360 | Other disorders of the kidney and ureters | genitourinary |
| 758 | -1.052 | -1.936 | -0.169 | 1.96E-02 | 49 | 43335 | Chromosomal anomalies and genetic disorders | congenital anomalies |
| 362.2 | -0.176 | -0.325 | -0.027 | 2.03E-02 | 2001 | 35116 | Degeneration of macula and posterior pole of retina | sense organs |
| 741.4 | 0.361 | 0.056 | 0.667 | 2.05E-02 | 405 | 40626 | Joint effusions | musculoskeletal |
| 447 | 0.279 | 0.041 | 0.517 | 2.15E-02 | 678 | 35748 | Other disorders of arteries and arterioles | circulatory system |
| 681.3 | 0.272 | 0.040 | 0.504 | 2.15E-02 | 712 | 29930 | Cellulitis and abscess of arm/hand | dermatologic |
| 365.2 | 0.316 | 0.045 | 0.586 | 2.22E-02 | 525 | 35664 | Primary angle-closure glaucoma | sense organs |
| 180 | -0.311 | -0.578 | -0.044 | 2.23E-02 | 546 | 20060 | Cervical cancer and dysplasia | neoplasms |
| 575.9 | 0.886 | 0.123 | 1.650 | 2.29E-02 | 64 | 39220 | Nonspecific abnormal findings on radiological and other examination of biliary tract | digestive |
| 636.3 | -0.363 | -0.678 | -0.047 | 2.42E-02 | 424 | 24045 | Hemorrhage in early pregnancy | pregnancy complications |
| 722.1 | -0.107 | -0.200 | -0.014 | 2.43E-02 | 5088 | 29987 | Displacement of intervertebral disc | musculoskeletal |
| 411.4 | 0.093 | 0.012 | 0.175 | 2.45E-02 | 8554 | 32289 | Coronary atherosclerosis | circulatory system |
| 525.2 | 0.799 | 0.101 | 1.498 | 2.50E-02 | 77 | 36834 | Atrophy of edentulous alveolar ridge | digestive |
| 761 | -0.095 | -0.179 | -0.012 | 2.53E-02 | 6415 | 34222 | Cervicalgia | symptoms |
| 442.8 | 0.660 | 0.080 | 1.240 | 2.58E-02 | 111 | 35748 | Aneurysm of other specified artery | circulatory system |
| 447.1 | 0.563 | 0.066 | 1.061 | 2.65E-02 | 150 | 35748 | Stricture of artery | circulatory system |
| 931 | -0.202 | -0.381 | -0.023 | 2.67E-02 | 1234 | 23549 | Contact dermatitis and other eczema due to plants [except food] | dermatologic |
| 433 | 0.104 | 0.012 | 0.196 | 2.69E-02 | 5538 | 37400 | Cerebrovascular disease | circulatory system |
| 871.3 | 0.421 | 0.048 | 0.794 | 2.69E-02 | 272 | 34273 | Open wound of foot except toe(s) alone | injuries & poisonings |
| 348.2 | 0.502 | 0.053 | 0.951 | 2.83E-02 | 189 | 36783 | Cerebral edema and compression of brain | neurological |
| 454.1 | -0.179 | -0.340 | -0.019 | 2.88E-02 | 1546 | 28627 | Varicose veins of lower extremity | circulatory system |
| 173 | -0.106 | -0.202 | -0.011 | 2.97E-02 | 5003 | 33378 | Neoplasm of uncertain behavior of skin | neoplasms |
| 442.1 | 0.174 | 0.016 | 0.332 | 3.12E-02 | 1659 | 35748 | Aortic aneurysm | circulatory system |
| 530.13 | -0.237 | -0.453 | -0.020 | 3.19E-02 | 824 | 21202 | Barrett's esophagus | digestive |
| 348.9 | 0.626 | 0.053 | 1.200 | 3.21E-02 | 115 | 36783 | Other conditions of brain, NOS | neurological |
| 795.8 | -1.003 | -1.922 | -0.084 | 3.25E-02 | 44 | 44805 | Abnormal tumor markers | symptoms |
| 200.1 | -0.485 | -0.930 | -0.040 | 3.25E-02 | 189 | 38350 | Polycythemia vera | neoplasms |
| 565.1 | 0.598 | 0.048 | 1.147 | 3.30E-02 | 123 | 37824 | Anal and rectal polyp | digestive |
| 694 | 0.286 | 0.021 | 0.550 | 3.45E-02 | 542 | 34329 | Dyschromia and Vitiligo | dermatologic |
| 560.3 | 1.009 | 0.070 | 1.948 | 3.52E-02 | 42 | 28963 | Peritoneal or intestinal adhesions | digestive |
| 626.8 | 0.347 | 0.023 | 0.670 | 3.58E-02 | 424 | 9030 | Infertility, female | genitourinary |
| 705.1 | -0.781 | -1.512 | -0.051 | 3.59E-02 | 71 | 35569 | Dyshidrosis | dermatologic |
| 643 | -0.564 | -1.090 | -0.037 | 3.60E-02 | 144 | 25579 | Excessive vomiting in pregnancy | pregnancy complications |
| 627.1 | 0.193 | 0.012 | 0.373 | 3.63E-02 | 1348 | 9030 | Postmenopausal bleeding | genitourinary |
| 202 | 0.327 | 0.020 | 0.635 | 3.71E-02 | 396 | 43470 | Cancer of other lymphoid, histiocytic tissue | neoplasms |
| 741 | 0.181 | 0.011 | 0.352 | 3.72E-02 | 1329 | 40626 | Symptoms and disorders of the joints | musculoskeletal |
| 433.3 | 0.148 | 0.008 | 0.288 | 3.80E-02 | 2094 | 37400 | Cerebral ischemia | circulatory system |
| 361.1 | -0.364 | -0.708 | -0.020 | 3.80E-02 | 318 | 35664 | Retinal detachment with retinal defect | sense organs |
| 256.4 | 0.290 | 0.016 | 0.563 | 3.82E-02 | 583 | 21751 | Polycystic ovaries | endocrine/metabolic |
| 694.2 | 0.324 | 0.017 | 0.631 | 3.83E-02 | 402 | 34329 | Other dyschromia | dermatologic |
| 256 | 0.278 | 0.013 | 0.544 | 3.98E-02 | 615 | 21751 | Ovarian dysfunction | endocrine/metabolic |
| 202.2 | 0.326 | 0.015 | 0.638 | 4.02E-02 | 385 | 43470 | Non-Hodgkins lymphoma | neoplasms |
| 110.12 | 0.227 | 0.010 | 0.444 | 4.07E-02 | 814 | 29383 | Althete's foot | infectious diseases |
| 253.2 | 0.718 | 0.030 | 1.406 | 4.07E-02 | 80 | 38173 | Pituitary hypofunction | endocrine/metabolic |
| 550.4 | 0.213 | 0.009 | 0.417 | 4.08E-02 | 927 | 36398 | Umbilical hernia | digestive |
| 601.1 | -0.228 | -0.446 | -0.009 | 4.09E-02 | 905 | 9958 | Prostatitis | genitourinary |
| 875 | 0.581 | 0.023 | 1.139 | 4.11E-02 | 121 | 34273 | Non-healing surgical wound | injuries & poisonings |
| 433.31 | 0.145 | 0.004 | 0.286 | 4.32E-02 | 2061 | 37400 | Transient cerebral ischemia | circulatory system |
| 286.2 | 0.086 | 0.003 | 0.170 | 4.35E-02 | 6889 | 34189 | Encounter for long-term (current) use of anticoagulants | hematopoietic |
| 440.1 | 0.334 | 0.009 | 0.658 | 4.40E-02 | 362 | 35748 | Atherosclerosis of renal artery | circulatory system |
| 316 | -0.210 | -0.414 | -0.005 | 4.44E-02 | 960 | 32208 | Substance addiction and disorders | mental disorders |
| 574.1 | -0.141 | -0.278 | -0.003 | 4.47E-02 | 2088 | 39220 | Cholelithiasis | digestive |
| 240 | 0.198 | 0.004 | 0.391 | 4.56E-02 | 1030 | 31072 | Simple and unspecified goiter | endocrine/metabolic |
| 585.1 | 0.140 | 0.003 | 0.277 | 4.56E-02 | 2218 | 33360 | Acute renal failure | genitourinary |
| 287.32 | 0.406 | 0.005 | 0.806 | 4.71E-02 | 237 | 34189 | Secondary thrombocytopenia | hematopoietic |
| 573.3 | -0.493 | -0.980 | -0.006 | 4.75E-02 | 159 | 34148 | Hepatomegaly | digestive |
| 414 | 0.135 | 0.001 | 0.268 | 4.76E-02 | 2485 | 32289 | Other forms of chronic heart disease | circulatory system |
| 157 | -0.640 | -1.275 | -0.005 | 4.81E-02 | 92 | 41789 | Pancreatic cancer | neoplasms |

Urate-PRS derived from GWAS of European ancestry. Bonferroni significant phecodes were emphasized in bold.

## Supplementary table 5. Results of PRS-wide association with gout

| PRS | beta | L95 | U95 | p-value |
| --- | --- | --- | --- | --- |
| Psoriatic arthritis | 0.020 | -0.024 | 0.064 | 3.81E-01 |
| Rheumatoid arthritis | 0.053 | 0.007 | 0.099 | 2.44E-02 |
| Spondyloarthropathy | 0.021 | -0.049 | 0.090 | 5.61E-01 |
| BMI | 0.244 | -0.064 | 0.551 | 1.20E-01 |
| LDL | 0.033 | -0.133 | 0.198 | 7.00E-01 |
| CHD | **0.928** | **0.446** | **1.410** | **1.63E-04** |
| Atrial fibrillation | **1.040** | **0.570** | **1.509** | **1.42E-05** |
| T2D | **0.733** | **0.368** | **1.097** | **8.17E-05** |
| Breast Cancer | -0.198 | -0.394 | -0.001 | 4.88E-02 |
| Prostate Cancer | 0.452 | 0.111 | 0.793 | 9.37E-03 |

PRS: polygenic risk score; BMI: body mass index; LDL: low density lipoprotein cholesterol; T2D: type 2 diabetes; CHD: coronary heart disease. Bonferroni significant PGSs were emphasized in bold.

## Supplementary table 6. Odds ratio across PRS quintiles

| Bin | Quintile | OR | L95 | U95 | p-value | Case N | Total N | Prevalence (%) |
| --- | --- | --- | --- | --- | --- | --- | --- | --- |
| Bin1 | (-1.97, -1.43] | 0.31 | 0.13 | 0.77 | 1.15e-2 | 5 | 230 | 2.2 |
| Bin2 | (-1.43, -0.892] | 0.53 | 0.46 | 0.60 | 1.28e-20 | 268 | 7803 | 3.4 |
| Bin3 (reference) | (-0.892, -0.357] | 1 | 1 | 1 | / | 1598 | 26393 | 6.1 |
| Bin4 | (-0.357, 0.179] | 1.96 | 1.79 | 2.13 | 4.79e-52 | 1004 | 9306 | 10.8 |
| Bin5 | (0.179, 0.717] | 3.23 | 2.35 | 4.44 | 4.52e-13 | 54 | 330 | 16.4 |

## Supplementary table 7. Prediction of gout status in the testing dataset using combined model

| Predicted | Diagnosed | |
| --- | --- | --- |
|  | **Gout** | **No gout** |
| Gout | 389 | 2844 |
| No Gout | 172 | 8759 |

**Reference:**

Tin, A., Marten, J., Halperin Kuhns, V.L., Li, Y., Wuttke, M., Kirsten, H., et al. (2019). Target genes, variants, tissues and transcriptional pathways influencing human serum urate levels. *Nat Genet* 51(10)**,** 1459-1474. doi: 10.1038/s41588-019-0504-x.
